# Supplementary material for: Biophysically inspired mean-field model of neuronal populations driven by ion-exchange mechanisms
Source: eLife. 2026 Sep 30;14:RP104249. doi: 10.7554/eLife.104249 (PMC13626649; doi:10.7554/eLife.104249)
Supplement: Supplementary file 2 [file elife-104249-supp1.docx]

**Supplementary File 1. List of parameters’ values used for each simulation in the manuscript.** If not present, the parameter is not applicable for that case, or different values have been used, as specified in the corresponding caption.

|  | **Fig. 2a** | **Fig. 3a** | **Fig. 4a-b** | **Fig. 4c** | **Fig. 5** | **Fig. 5c** | **Fig. 6b-c** |
| --- | --- | --- | --- | --- | --- | --- | --- |
| **Δ** | - | 1 | 1 | 1 | 1 | 1 | 1 |
| **J** | - | 1 | 1 | 0.01 | 0.08 | 2 | 0.08 |
| **η** | - | 0 | 0 | 0 | 0 | 0 | 0 |
| **K_bath** | - | - | 15.5 | 15.5 | - | 15.5 | - |
| **c₋** | - | -44.24 | - | -44.24 | -44.24 | -44.24 | -44.24 |
| **c₊** | - | -20.40 | - | -20.40 | -20.40 | -20.40 | -20.40 |
| **R₋** | - | 0.34 | - | 0.001 | 0.02 | 0.50 | 0.02 |
| **R₊** | - | -0.38 | - | -0.01 | -0.1 | -0.50 | -0.1 |
| **C_m** | 1 | 1 | 10 | 16 | 1 | 1 | 1 |
| **τ_n** | 4 | 4 | 2.4 | 8 | 4 | 4 | 4 |
| **γ** | 0.04 | 0.04 | 0.04 | 0.00025 | 0.05 | 0.04 | 0.04 |
| **ε** | 0.001 | 0.001 | 0.001 | 0.0001 | 0.001 | 0.001 | 0.001 |
| **x(t=0)** | - | - | 0.17 | - | 0.1 | 0.10 | 0.10 |
| **V(t=0)** | -5 | -15 | -81.78 | -15 | -15 | -15 | -15 |
| **n(t=0)** | 0.40 | 0.45 | 0.03 | 0.05 | 0.45 | 0.45 | 0.45 |
| **ΔK_i(t=0)** | -0.10 | -3.50 | -4.92 | -0.23 | - | -5 | -5 |
| **K_g(t=0)** | 3 | -12 | -14.88 | 7.93 | - | -16 | -16 |
| **N** | 1 | 3000 | 3000 | - | - | - | - |
