# Supplementary material for: Biophysically inspired mean-field model of neuronal populations driven by ion-exchange mechanisms
Source: eLife. 2026 Sep 30;14:RP104249. doi: 10.7554/eLife.104249 (PMC13626649; doi:10.7554/eLife.104249)
Supplement: Peer review history

# Editor report: eLife Assessment

**DOI:** 10.7554/eLife.104249.3.sa0

**Authors:** Tatyana O Sharpee

**Affiliations:** Salk Institute for Biological Studies United States

**Keywords:** Incomplete, Useful

This manuscript presents a **useful** mean-field model for a network of Hodgkin-Huxley neurons retaining the equations for ion exchange between the intracellular and extracellular space. The mean-field model derived in this work relies on approximations and heuristic arguments that, on the one hand, allow a closed-form derivation of the mean-field equations, but also raise questions about their justifications and the degree to which the results agree with experiments as well as direct numerical simulations. While the revised manuscript is much improved, reviewers continue to question the methodology for reducing model dimensionality and therefore the evidence for the utility of this approach remains **incomplete** at present.

# Referee report: Reviewer #1 (Public review)

**DOI:** 10.7554/eLife.104249.3.sa1

Summary:

In this manuscript the authors derive a mean-field model for a network of Hodgkin-Huxley neurons retaining the equations for ion exchange between the intracellular and extracellular space.

The mean-field model derived in this work relies on approximations and heuristic arguments that, on the one hand, allow a closed-form derivation of the mean-field equations, and on the other hand restrict its validity to a limited regime of activity corresponding to quasi-synchronous neuronal populations. Therefore, rather than an exact mean-field representation, the model provides a description of a mesoscopic population of connected neurons driven by ion exchange dynamics.

Strengths:

The idea of deriving a mean-field model which relates the slow-timescale biophysical mechanism of ion exchange and transportation in the brain to the fast-timescale electrical activities of large neuronal ensembles.

Weaknesses:

The idea underlying this work is not completely implemented in practice.

The derived mean field model do not show a one-to-one correspondence with the neural network simulations, except in strongly synchronous regimes. The agreement with the in vitro experiment is hardly evident, both for the mean-field model and for the network model. The assumptions made to derive the closed-form equations of the mean field model have not been justified by any biological reason, they just allow for the mathematical derivation. The final form of the mean-field equations do not clarify whether or not microscopic variables are used together with macroscopic variables in an inconsistent mixture.

Comments on revisions:

The main weaknesses I listed in the first report are still present, since the authors did not answer my questions on a solid basis. I report the list for completeness:

(1) It seems that the reduction methodology that is employed is not the most suitable one for the single-neuron model they are considering.

(2) The formulation of the mean-field derivation is unnecessarily complicated. It could be heavily simplified by following previously published approaches to derive biologically realistic neural masses.

(3) The model seems to work only for highly synchronized situations and not for the standard asynchronous evolution usually observed in neural circuits.

Therefore, my statement remains unchanged.

# Referee report: Reviewer #2 (Public review)

**DOI:** 10.7554/eLife.104249.3.sa2

Summary:

The authors aiming in developing a neural mass model characterized by few collective variables mimicking the dynamics of a network of Hodgkin - Huxley neurons encompassing ion-exchange mechanisms. They describe in details the derivation of the mean-field model , then they compare experimental results obtained for the hippocampus of a mice with the neural network simulations and the mean-field results. Furthermore, they report a bifurcation analysis of the developed model and simulation of a small network containing various coupled neural masses, somehow moving towards the simulation of an entire connectome.

Strengths:

The author attempts to develop a mean-field model for a globally coupled network of heterogeneous Hodgkin-Huxley neurons with explicit ion exchange mechanism between the cell interior and exterior.

Weaknesses:

(1) They do not employ the reduction methodology more suited for the single neuron model they consider.

(2) Their derivation of the neural mass model is based on several assumptions, and not all well justified.

(3) Their formulation of the mean-field derivation is unnecessary complicated, it can be strongly simplified by following previously published approaches to derive biologically realistic neural masses.

(4) Their model seems to work only for highly synchronized situations and not for the standard asynchronous evolution usually observed in neural circuits.

General Statements:

The authors honestly declared the many limitations of their approach, once assumed this the results of the mean-field are somehow inconsistent with the neural network simulations as expected.

The authors suggest to employ this model for the simulations on the whole connectome to follow seizure propagation, however I believe that a simpler model, as the Epileptor, remains superior in this respect to this model. That indeed includes biophysical parameters but their correspondence with the ones employed in the network dynamics remain elusive, due to the many assumptions required to derive this mean field model. Furthermore it is more complicated than the Epileptor, I do not think that the present model will be largely employed by the community.

Comments on revisions:

The authors have corrected mistakes present in the manuscript and put a correct list of references.

However, they refuse

(1) To simplify the formulation of the model, the model contains unnecessary complications, as I have clearly written in my report, the authors agree, but they do not want to change the formulation;

(2) To derive the mean field model in a simpler way, as possible, and as I asked many times in my Referee report, this would help the readers to understand the important aspect of the derivation, without not needed and confusing complicated formulations;

(3) To compare direct simulations of the network with neural mass results in sub-section "Bifurcation analysis: emergent network states and multistability" to show bistability, as I asked.

As a matter of fact the performed modifications do not solve my previous doubts on the validity of the results reported in the manuscript.

Therefore, my previous assessments remain valid.

# Author comment: Author response

**DOI:** 10.7554/eLife.104249.3.sa3

**Authors:** Giovanni Rabuffo, Abhirup Bandyopadhyay, Carmela Calabrese, Kashyap Gudibanda, Damien Depannemaecker, Lavinia Mitiko Takarabe, Sourin Chatterjee, Maria Luisa Saggio, Mathieu Desroches, Anton Ivanov, Marja-Leena Linne, Christophe Bernard, Spase Petkoski, Viktor Jirsa

**Affiliations:** Aix-Marseille Université Marseille France; University of Amsterdam Amsterdam Netherlands; Aix-Marseille Université Marseille France; Aix-Marseille Université Marseille France; Aix-Marseille Université Marseille France; Aix-Marseille Université Marseille France; Aix-Marseille Université Marseille France; Aix-Marseille Université Marseille France; Research Centre Inria Sophia Antipolis – Méditerranée Montpellier France; Aix-Marseille University, Inserm UMR1106 Marseille, France; Faculty of Medicine and Health Technology, Tampere University Tampere Finland; Aix-Marseille Université, Marseille Marseille France; Institut de Neurosciences des Systèmes Marseille France; Aix-Marseille Université Marseille France

The following is the authors’ response to the current reviews.

**Reviewer #1 (Public review)**

Summary:

In this manuscript the authors derive a mean-field model for a network of Hodgkin-Huxley neurons retaining the equations for ion exchange between the intracellular and extracellular space.

The mean-field model derived in this work relies on approximations and heuristic arguments that, on the one hand, allow a closed-form derivation of the mean-field equations, and on the other hand restrict its validity to a limited regime of activity corresponding to quasi-synchronous neuronal populations. Therefore, rather than an exact mean-field representation, the model provides a description of a mesoscopic population of connected neurons driven by ion exchange dynamics.

Strengths:

The idea of deriving a mean-field model which relates the slow-timescale biophysical mechanism of ion exchange and transportation in the brain to the fast-timescale electrical activities of large neuronal ensembles.

Weaknesses:

The idea underlying this work is not completely implemented in practice.

The derived mean field model do not show a one-to-one correspondence with the neural network simulations, except in strongly synchronous regimes. The agreement with the in vitro experiment is hardly evident, both for the mean-field model and for the network model. The assumptions made to derive the closed-form equations of the mean field model have not been justified by any biological reason, they just allow for the mathematical derivation. The final form of the mean-field equations do not clarify whether or not microscopic variables are used together with macroscopic variables in an inconsistent mixture.

Comments on revisions:

The main weaknesses I listed in the first report are still present, since the authors did not answer my questions on a solid basis. I report the list for completeness:

(1) It seems that the reduction methodology that is employed is not the most suitable one for the single-neuron model they are considering.

(2) The formulation of the mean-field derivation is unnecessarily complicated. It could be heavily simplified by following previously published approaches to derive biologically realistic neural masses.

(3) The model seems to work only for highly synchronized situations and not for the standard asynchronous evolution usually observed in neural circuits.

Therefore, my statement remains unchanged.

**Reviewer #2 (Public review)**

Summary:

The authors aiming in developing a neural mass model characterized by few collective variables mimicking the dynamics of a network of Hodgkin - Huxley neurons encompassing ion-exchange mechanisms. They describe in details the derivation of the mean-field model , then they compare experimental results obtained for the hippocampus of a mice with the neural network simulations and the mean-field results. Furthermore, they report a bifurcation analysis of the developed model and simulation of a small network containing various coupled neural masses, somehow moving towards the simulation of an entire connectome.

Strengths:

The author attempts to develop a mean-field model for a globally coupled network of heterogeneous Hodgkin-Huxley neurons with explicit ion exchange mechanism between the cell interior and exterior.

Weaknesses:

(1) They do not employ the reduction methodology more suited for the single neuron model they consider.

(2) Their derivation of the neural mass model is based on several assumptions, and not all well justified.

(3) Their formulation of the mean-field derivation is unnecessary complicated, it can be strongly simplified by following previously published approaches to derive biologically realistic neural masses.

(4) Their model seems to work only for highly synchronized situations and not for the standard asynchronous evolution usually observed in neural circuits.

General Statements:

The authors honestly declared the many limitations of their approach, once assumed this the results of the mean-field are somehow inconsistent with the neural network simulations as expected.

The authors suggest to employ this model for the simulations on the whole connectome to follow seizure propagation, however I believe that a simpler model, as the Epileptor, remains superior in this respect to this model. That indeed includes biophysical parameters but their correspondence with the ones employed in the network dynamics remain elusive, due to the many assumptions required to derive this mean field model. Furthermore it is more complicated than the Epileptor, I do not think that the present model will be largely employed by the community.

Comments on revisions:

The authors have corrected mistakes present in the manuscript and put a correct list of references.

However, they refuse

(1) To simplify the formulation of the model, the model contains unnecessary complications, as I have clearly written in my report, the authors agree, but they do not want to change the formulation;

(2) To derive the mean field model in a simpler way, as possible, and as I asked many times in my Referee report, this would help the readers to understand the important aspect of the derivation, without not needed and confusing complicated formulations;

(3) To compare direct simulations of the network with neural mass results in sub-section "Bifurcation analysis: emergent network states and multistability" to show bistability, as I asked.

As a matter of fact the performed modifications do not solve my previous doubts on the validity of the results reported in the manuscript.

Therefore, my previous assessments remain valid.

We thank the editors and the two reviewers for their continued engagement with our manuscript. The three weaknesses retained from the first round are essentially identical between the two public reviews:

(i) The reduction methodology is not the most suitable for the single-neuron model we consider;

(ii) The mean-field derivation is unnecessarily complicated;

(iii) The model works only in highly synchronous regimes and does not reproduce the asynchronous evolution typical of neural circuits.

Both reviewers explicitly note that their assessments remain unchanged and we have decided not to alter the formulation of the model. We use this response to state—on the public record—exactly where we agree with the reviewers, where we disagree, and why.

On point (i): the reduction methodology.

We fully agree with the reviewers' technical observation: the Ott–Antonsen / Lorentzian-ansatz reduction in the form introduced by Montbrió, Pazó and Roxin (2015) is exact for canonical Type I neurons (QIF), whose membrane-potential equation is quadratic, and is not directly applicable to a Type II / Hodgkin–Huxley-type neuron whose voltage dynamics is cubic-like. On this point there is no disagreement.

Where we differ is in the conclusion the reviewers draw from this observation. The reviewers read our work as applying an inappropriate reduction methodology to an inappropriate neuron model. We instead positioned our work, from the outset, as an extension of that methodology: we keep the biophysically detailed Hodgkin–Huxley substrate (because it is the only level at which extracellular ion concentrations, depolarization block, bursting and seizure-like events are biophysically grounded), and we adapt the reduction by approximating the cubic voltage nullcline as a piece-wise quadratic with two parabolas of opposite curvature. This is explicitly an approximate, not exact, mean-field. The Lorentzian ansatz is then applied on each branch of the piece-wise quadratic, with the limitations of this construction analyzed in the manuscript.

The reviewers' alternative—starting from a Type I canonical model and grafting on biophysical features—would indeed yield an exact mean-field, but it would forfeit precisely what motivates our work: a tractable mesoscopic description in which the slow variables are physiologically interpretable ion concentrations rather than phenomenological parameters. The trade-off is that we give up exact rigour in order to construct a bridge between the Montbrió-style next-generation neural mass models on one side and the Epileptor on the other, with the additional benefit that the parameters of the resulting neural mass retain a biophysical correspondence (e.g., [K^+^]_bath, Δ[K^+^]_int, [K^+^]_g, the gating variable n) that the Epileptor does not afford.

We therefore respectfully maintain our position: the methodology is not "the wrong reduction for a Type II neuron"; it is an extended reduction designed to be applicable beyond the Type I case, with explicitly characterized validity.

On point (ii): the formulation is unnecessarily complicated.

We agree with the reviewers that, given the assumptions we ultimately adopt, namely that the gating variable n and the potassium concentrations Δ[K^+^]_int and [K^+^]_g are treated as collective (mesoscopic) variables shared by the population, with n a function of the average membrane potential, the closed neural mass equations could be reached by the more direct path used by Guerreiro et al. (2022) and the related literature (R1–R7). In the revised manuscript we now state this explicitly, and we note that the same five-dimensional system arises under either derivation.

Our choice to follow Chen and Campbell (2022) is motivated by the fact that it makes each approximation visible at the point where it is invoked. In particular, it exposes the moment-closure step (Eq. 19), the vanishing-flux boundary condition (Eq. 28), and the locations where microscopic and mesoscopic variables enter the description. We believe that for a reader trying to extend our framework, for instance to a setting with partial heterogeneity in the slow variables, or with stochastic gating, this is the more useful presentation. We have added a remark stating that the simpler Guerreiro-type derivation reaches the same equations under our assumptions, so that readers can take whichever route they find clearer.

On point (iii): the model only works in highly synchronous regimes.

Here we partially agree and partially disagree, and we would like the partial disagreement to appear on the public record.

We agree that the Lorentzian ansatz is, strictly, valid in regimes where the population's membrane potential distribution is unimodal, that is, when essentially all neurons sit on the same side of the threshold V*. Where we disagree is with the implication that the mean-field model fails outside the strongly synchronous regime. The supplementary analysis in Fig. S2, added in the previous round, quantifies the error introduced by the first-moment approximation of n as a collective variable across the full range of [K^+^]_bath values, spanning quiescent, bursting, seizure-like, sustained ictal and depolarization-block dynamics. The fraction of neurons whose gating variable deviates from the population mean is below 2% for the parameters used throughout the manuscript, and the error becomes appreciable only during the brief transitions between sub- and supra-threshold states. These are precisely the moments at which the population is genuinely bimodal and the single-Lorentzian assumption is theoretically expected to leak. In other words, the error peaks coincide with the moments where our derivation tells us in advance that the assumption is locally invalid; the model "knows where it fails." Away from these transitions, the mean-field tracks the population average across all dynamical regimes shown in Fig. 3, not only in the most strongly synchronized ones.

This is, in our view, the strongest argument we can make: we are not claiming exactness, and we are not unaware of the limitations. We have characterized them analytically (the construction of the piece-wise Lorentzian, and the theoretical reason a closed solution exists only when the two branches collapse onto one), and we have characterized them numerically (Fig. S2). The deviations are bounded, their location in parameter space is well identified, and they coincide with transitions where the underlying assumption is locally violated. We believe this constitutes a controlled approximation rather than an uncontrolled one, and we would like this distinction to be visible to readers of the Reviewed Preprint.

We note, in this connection, that the reviewers' preferred reference point, the next-generation neural mass model of Montbrió et al. (2015), which is exact and one-to-one with its underlying network, is exact precisely because the underlying network is a network of QIF neurons. The corresponding statement for a network of Hodgkin–Huxley-type neurons with explicit ion exchange does not, to our knowledge, exist in closed form, and may not exist at all. The relevant question is therefore not whether our model matches the exactness of the QIF case, but whether the controlled approximation we provide is useful. Given the qualitative agreement with neural-network simulations across the full range of [K^+^]_bath, the qualitative agreement with the in vitro recordings, and the recovery of the expected bifurcation structure with new emergent regimes, we believe the answer is yes.

Other outstanding points in the review.

Reviewer 2 reiterates the view that the Epileptor remains superior for whole-connectome seizure-propagation simulations because it is simpler and better characterized. We do not dispute that the Epileptor is more thoroughly analyzed and more parsimonious. The complementarity we propose is not a replacement but a parameter-grounding, as the Epileptor's phenomenological parameters (excitability, slow permittivity) acquire, in the present framework, an interpretation in terms of measurable biophysical quantities (extracellular potassium, intracellular potassium variation, glial buffering).

We thank the reviewers and editors once again for their careful reading, and we are grateful that the points of disagreement have been sharpened to a state where readers can judge them transparently.

The following is the authors’ response to the original reviews.

**Public Reviews:**

**Reviewer #1 (Public review):**

Summary:

In this manuscript, the authors derive a mean-field model for a network of Hodgkin-Huxley neurons retaining the equations for ion exchange between the intracellular and extracellular space.

The mean-field model derived in this work relies on approximations and heuristic arguments that, on the one hand, allow a closed-form derivation of the mean-field equations, and on the other hand restrict its validity to a limited regime of activity corresponding to quasi-synchronous neuronal populations. Therefore, rather than an exact mean-field representation, the model provides a description of a mesoscopic population of connected neurons driven by ion exchange dynamics.

We agree with the reviewer's characterization. Our manuscript describes the derivation as relying on "approximations and heuristic arguments" and states that "the derivation is not exact"; what we provide is a controlled, approximate mesoscopic description in which the slow variables are physiologically interpretable ion concentrations rather than phenomenological parameters. An exact closed-form thermodynamic limit is, to our knowledge, available only for canonical Type I (QIF) networks (Montbrió, Pazó and Roxin, 2015) and a few of their extensions; it is not currently known for a Hodgkin–Huxley-type network with explicit ion-exchange dynamics. We acknowledge that the original description of the regime of validity may have caused confusion on this point, and in the revised manuscript we have therefore replaced the looser formulation "strongly synchronous regimes" by the more accurate "regimes where the membrane-potential distribution is unimodal and can be reasonably approximated by a Lorentzian" throughout the manuscript.

Strengths:

The idea of deriving a mean-field model that relates the slow-timescale biophysical mechanism of ion exchange and transportation in the brain to the fast-timescale electrical activities of large neuronal ensembles.

We thank the reviewer for recognizing the motivation behind our work. This explicit coupling between slow biophysical ion dynamics and fast electrical activity is precisely the feature we tried to preserve in the reduction, even at the cost of giving up exactness.

Weaknesses:

The idea underlying this work is not completely implemented in practice.

We address this general statement through the four specific sub-points the reviewer raises in the paragraph that follows.

The derived mean field model does not show a one-to-one correspondence with the neural network simulations, except in strongly synchronous regimes.

We partially agree and partially disagree. We agree that the Lorentzian ansatz is strictly valid where the membrane-potential distribution is unimodal, i.e. when essentially all neurons sit on the same side of the threshold V*. We disagree with the implication that the mean-field fails outside this regime. To make this claim quantitative, we added a new supplementary figure (Fig. S2) that quantifies the deviation of individual neurons' gating variables from the population mean across the full range of [K^+^]_bath values—quiescent, bursting, seizure-like, sustained ictal and depolarization-block dynamics. The fraction of deviating neurons is below 2% for the parameters used in the manuscript, with localized peaks only during the brief, genuinely bimodal transitions between sub- and supra-threshold states—precisely the moments at which the theory predicts the assumption to be locally invalid. Away from these transitions, the mean-field tracks the population average across all dynamical regimes shown in Fig. 3, not only in the strongly synchronized ones.

The agreement with the in vitro experiment is hardly evident, both for the mean-field model and for the network model.

We acknowledge that the experimental and simulated traces in the original Fig. 4 did not match quantitatively; this was never our intention. The figure and its caption have been reorganized in the revised manuscript to frame the comparison as qualitative: we aim to demonstrate the shared structure i.e., the slow modulation of fast population activity by extracellular potassium fluctuations, rather than to claim a quantitative fit.

We also added two clarifications that account for the residual differences: (i) the network simulations were intentionally run with rescaled biophysical parameters (membrane capacitance, gating time constants) to keep the computational cost feasible, a standard practice when the goal is to validate dynamical mechanisms rather than absolute timescales; (ii) the in vitro LFP recordings were AC-coupled, so the slow DC components visible in the mean-field traces are filtered out at acquisition.

The assumptions made to derive the closed-form equations of the mean-field model have not been justified by any biological reason, they just allow for the mathematical derivation.

We agree that the modelling assumptions were scattered through the original derivation. In the revised manuscript, the three core assumptions are stated explicitly at the point of derivation: (i) the gating variable n is treated as a collective, population-averaged variable; (ii) the potassium concentrations Δ[K^+^]_int and [K^+^]_g are homogeneous across the population, biophysically justified by the rapid redistribution of ions through diffusion and electrochemical gradients, which enforces near-instantaneous equilibration at the mesoscopic scale; (iii) no heterogeneity is assumed at the level of ion dynamics. The meaning of "locally homogeneous" is now defined explicitly.

On the biophysical motivation of the in vitro perturbation used in the experiment, we have added a new Methods subsection that explains how low extracellular Mg^2+^ unblocks NMDARs and abolishes the divalent-cation stabilisation of the resting membrane potential, depolarising hippocampal neurons and increasing the driving force for outward K^+^ currents. This provides a biophysical link between the experimental perturbation and the model's main control parameter, the extracellular potassium concentration. We also added a reference to the well-established model of epileptic discharges that underpins the experiment.

The final form of the mean-field equations does not clarify whether or not microscopic variables are used together with macroscopic variables in an inconsistent mixture.

We now explicitly acknowledge that in the spiking-network simulations the gating variable n is microscopic (each neuron has its own n_i), whereas in the mean-field derivation it is treated as mesoscopic and shared by the population. This asymmetry between modalities is discussed both in the Results and in the Limitations sections, and is identified as a likely source of some of the discrepancy between the two modalities.

We have also made the notation in Eqs. (36)–(37) consistent (firing rate r used throughout, full current-based dV/dt̄ restored) and fixed the typos and broken equation/reference labels that contributed to the impression of inconsistency (Eqs. 18, 28, 29; the Fig. 2(c) [K^+^] bath label; the lost reference at line 696).

**Reviewer #2 (Public review):**

Summary:

The authors aim to develop a neural mass model characterized by a few collective variables mimicking the dynamics of a network of Hodgkin – Huxley neurons encompassing ion-exchange mechanisms. They describe in detail the derivation of the mean-field model, then they compare experimental results obtained for the hippocampus of a mouse with the neural network simulations and the mean-field results. Furthermore, they report a bifurcation analysis of the developed model and simulation of a small network containing various coupled neural masses, somehow moving towards the simulation of an entire connectome.

We thank the reviewer for the accurate summary of the manuscript's structure and aims.

Strengths:

The author attempts to develop a mean-field model for a globally coupled network of heterogeneous Hodgkin-Huxley neurons with an explicit ion exchange mechanism between the cell interior and exterior.

We thank the reviewer for recognizing this objective. The retention of Hodgkin–Huxley dynamics with explicit ion exchange is precisely the feature that distinguishes our framework from QIF-based reductions, and it is what enables the slow variables of the resulting mean-field to retain a direct biophysical interpretation.

Weaknesses:

(1) It seems that the reduction methodology that is employed is not the most suitable one for the single-neuron model they are considering.

We agree, on technical grounds, with the observation: the Ott–Antonsen / Lorentzian-ansatz reduction is exact for canonical Type I neurons (QIF) and is not directly applicable to a Type II Hodgkin–Huxley-type neuron with a cubic-like voltage nullcline. Where we differ is in the conclusion. We did not apply an inappropriate reduction to an inappropriate neuron; we deliberately extended the methodology by approximating the cubic nullcline as a piece-wise quadratic with two parabolas of opposite curvature, and then applying the Lorentzian ansatz on each branch. The result is an explicitly approximate, biophysically grounded mean-field, with its regime of validity stated and quantified (Fig. S2).

To make this positioning explicit, we have added a paragraph to the Introduction that situates our work within the next-generation neural mass literature (Byrne et al. 2020; Montbrió, Pazó & Roxin 2015; Guerreiro et al. 2022; Forrester et al. 2024; Perl et al. 2023; Gerster et al. 2021; and works on short-term plasticity, adaptation, conductance-based reductions, spike-timing-dependent plasticity, random connectivity and noise) and clarifies that we see our contribution as complementary to these approaches, not as a competitor to the exact QIF reductions.

(2) The authors' derivation of the neural mass model is based on several assumptions, and not all well justified.

We agree that, in the original submission, the modelling assumptions were scattered through the derivation. In the revised manuscript, the three core assumptions are stated explicitly at the point of derivation: (i) the gating variable n is treated as a collective population-averaged variable; (ii) the potassium concentrations Δ[K^+^]_int and [K^+^]_g are homogeneous across the population, biophysically justified by the rapid redistribution of ions through diffusion and electrochemical gradients, which enforces near-instantaneous equilibration at the mesoscopic scale; (iii) no heterogeneity at the level of ion dynamics is assumed. The meaning of "locally homogeneous" is now defined explicitly. In addition, we have added Fig. S2, which quantifies numerically the error introduced by the moment-closure assumption (deviation below 2% for the parameters used in the manuscript).

(3) The formulation of the mean-field derivation is unnecessarily complicated. It could be heavily simplified by following previously published approaches to derive biologically realistic neural masses.

We agree that, under the assumptions ultimately adopted in our model—namely that n, Δ[K^+^]_int and [K^+^]_g are mesoscopic—the final five-dimensional system can be reached by the more direct path used by Guerreiro et al. (2022) and the related literature. We now state this explicitly in the revised manuscript and note that the same system arises under either derivation, so that the reader can take whichever route they find clearer. Our choice to retain the Chen and Campbell (2022) formalism is pedagogical: it exposes the moment-closure step (Eq. 19), the vanishing-flux boundary condition (Eq. 28), and the locations where microscopic versus mesoscopic variables enter the description, which is the more useful presentation for a reader wishing to extend the framework (e.g. to partial heterogeneity in the slow variables or to stochastic gating). We also made the notation in Eqs. (36)–(37) consistent (firing rate r used throughout, full current-based dV/dt̄ restored) and fixed a number of typos and broken equation/reference labels.

(4) The model seems to work only for highly synchronized situations and not for the standard asynchronous evolution usually observed in neural circuits.

We partially agree and partially disagree. We agree that the Lorentzian ansatz is strictly valid where the membrane-potential distribution is unimodal; we have replaced "strongly synchronous regimes" by this more accurate formulation throughout the manuscript. We disagree, however, with the implication that the mean-field is useful only in those regimes. Fig. S2, added in this revision, explicitly quantifies the deviation across all dynamical regimes (quiescent, bursting, seizure-like, sustained ictal and depolarization-block dynamics): it remains below 2% for the parameters used in the manuscript, with localized peaks only during the brief sub-to-supra-threshold transitions where the population is genuinely bimodal. Away from these transitions, the mean-field tracks the population average across all dynamical regimes shown in Fig. 3.

General Statements:

The authors honestly declared the many limitations of their approach. It is assumed that the results of the mean-field are somehow inconsistent with the neural network simulations as expected.

We thank the reviewer for acknowledging that the limitations are honestly declared. As detailed above and quantified in Fig. S2, the deviation from the network simulations is bounded and well characterized; it is not assumed but measured.

The authors suggest employing this model for the simulations on the whole connectome to follow seizure propagation, however, I believe that the Epileptor remains superior in this respect to this model. That indeed includes biophysical parameters but their correspondence with the ones employed in the network dynamics remains elusive, due to the many assumptions required to derive this mean-field model. Furthermore, it is more complicated than the Epileptor, I do not think that the present model will be largely employed by the community.

We do not propose our model as a direct replacement for the Epileptor and we do not dispute that the Epileptor is more thoroughly analyzed and more parsimonious. The complementarity we propose is not a replacement but a parameter-grounding: the Epileptor's phenomenological parameters (excitability, slow permittivity) acquire, in our framework, a concrete interpretation in terms of measurable biophysical variables (extracellular potassium, intracellular potassium variation, glial buffering). Retaining the Hodgkin–Huxley substrate is essential to ground these variables biophysically.

To make this complementarity more visible, the Limitations and Discussion section has been expanded to discuss the choice of a purely excitatory network as a first step (with excitatory–inhibitory generalizations available via the synaptic reversal potential) and to point to additional biological ingredients (calcium and other ions, plastic synapses, random connectivity and noise, adaptation, spike-timing-dependent plasticity) that the framework can accommodate, with reference to the next-generation neural mass literature.

We thank the reviewers and editors for their careful reading. We hope this public response makes our reasoning, the limits of our approach, and the concrete revisions made in this round transparent.

**Recommendations for the authors:**

**Reviewer #1 (Recommendations for the authors):**

(1) In general, the writing is scattered. Every time a model is introduced, one starts from the general formulation only to find that a very simplified case is used with respect to that formulation, which is very confusing. Authors need to reduce unnecessary formulations that confuse the reader and make it clear which formulations are actually used.

We thank the reviewer for this comment and understand the concern regarding the balance between general formulations and specific approximations. Our intention in including the more general equations and derivations (e.g., Eq. 7 and others) was pedagogical — to ensure completeness and transparency in the modeling steps, especially for readers less familiar with mean-field reductions of biophysically detailed models. These general forms also serve to clarify the assumptions underlying the simplifications we employ. In the latest version, we improved the clarity of core equations (e.g., Eq. 37), which form the basis of all simulations presented (see details below, in the answer to question 14).

(2) The Introduction would benefit from a wider view of the literature. The literature on exact mean field models (i.e. derived from the Lorentzian Ansatz) has flourished in the last years. In particular, it would be worth considering the following papers, where exact neural mass models are applied to perform whole-brain and large-scale brain simulations:

Forrester, M., Petros, S., Cattell, O., Lai, Y. M., O'Dea, R. D., Sotiropoulos, S., & Coombes, S. (2024). Whole brain functional connectivity: Insights from next generation neural mass modelling incorporating electrical synapses. PLOS Computational Biology, 20(12), e1012647.

Perl, Y. S., Zamora-Lopez, G., Montbrio, E., Monge-Asensio, M., Vohryzek, J., Fittipaldi, S.,

Campo, C. G., Moguilner, S., Ibanez, A., Tagliazucchi, E., Yeo, B. T. T., Kringelbach, M. L., & Deco, G. (2023). The impact of regional heterogeneity in whole-brain dynamics in the presence of oscillations. Network Neuroscience, 7(2), 632-660.

Byrne, Aine, James Ross, Rachel Nicks, and Stephen Coombes. "Mean-field models for EEG/MEG: from oscillations to waves." Brain topography 35, no. 1 (2022): 36-53.

Gerster, M., Taher, H., Skoch, A., Hlinka, J., Guye, M., Bartolomei, F.,... & Olmi, S. (2021). Patient-specific network connectivity combined with a next generation neural mass model to test clinical hypothesis of seizure propagation. Frontiers in Systems Neuroscience, 15, 675272.

Byrne, Aine, Reuben D. O'Dea, Michael Forrester, James Ross, and Stephen Coombes. "Next-generation neural mass and field modeling." Journal of neurophysiology 123, no. 2 (2020): 726-742.

Benitez-Stulz, Sophie, Samy Castro, Gregory Dumont, Boris Gutkin, and Demian Battaglia. "Compensating functional connectivity changes due to structural connectivity damage via modifications of local dynamics." bioRxiv (2024): 2024-05.

We have added the following paragraph:

“Recently, a class of these models, called next-generation neural mass models [42], has been developed based on an analytical approach introduced by [25] that allowed for the exact derivation of mean field parameters for a population of quadratic integrate-and-fire (QIF) neurons. These can be linked to EEG/MEG oscillations [43], including epipeltic seizures [43], and have been used to study various aspects of the whole-brain dynamics such as the low-dimensional manifold of the resting state [45,46], aging [47] and neural signatures of consciousness [48].”

We have also modified the preceding paragraph of the introduction that now reads:

“At the mesoscopic level, the observable properties of a neuronal ensemble are generally explained by statistical physics formalism of mean-field theory [19-22]. Mean-field models demonstrated a predictive value for studying the mesoscopic dynamics of neuronal populations [23], providing statistical descriptions of neuronal networks [2, 19, 24-29], which can be used to address questions related to network-level mechanisms [12, 24, 30].

In general, neural mass models have a low enough number of parameters to be tractable and provide general intuitions regarding mechanisms underlying complex neuronal activity [31-36]. For example, statistical population measures, such as the firing rate, can be used to assess mesoscopic dynamics [1, 7, 31, 36-41].”

(3) Moreover, conductance-based models have been already implemented in neural mass models not only in references [69, 71, 95], but also in:

Guerreiro, I. C., Di Volo, M., & Gutkin, B. (2023). A new generation of reduction methods for networks of neurons with complex dynamic phenotypes.

Capone, C., Di Volo, M., Romagnoni, A., Mattia, M., & Destexhe, A. (2019). State-dependent mean-field formalism to model different activity states in conductance-based networks of spiking neurons. Physical Review E, 100(6), 062413.

We have added the following sentence:

“Moreover, conductance-based couplings between the spiking neurons have been already implemented in neural mass models [58, 59, 91, 93, 121], but without an extracellular exchange mechanism.”

(4) Sec. 1.1 As previously established in the literature, a system of all-to-all coupled neuronal equations can be solved exactly in the thermodynamic limit (i.e., infinite neurons limit) if the single neuron membrane potential equation is a quadratic function and if the instantaneous distribution of membrane potentials of neurons in a population is described by a Lorentzian [Montbrió, E., Pazó, D. & Roxin, A. Physical Review X 5 (2), 021028 (2015)]. This means that the thermodynamic limit can be performed for a Canonical Type I model like the quadratic integrate-and-fire.

What is the biological justification and the reason to approximate a different neuron type (a type II neuron model), whose membrane potential equation resembles a cubic function, with a quadratic function? The fact that it can be solved in the quadratic approximation is not, in my opinion, a sufficient justification. It would be more correct to start from a type I neuron at the microscopic level with a quadratic function and then provide additional biological features.

We thank the reviewer for raising this important point. We respectfully disagree with the notion that starting from a canonical Type I model (such as the quadratic integrate-and-fire neuron) would be a more biologically grounded approach. While the quadratic form is analytically convenient, it does not capture certain key features of neuronal excitability particularly those related to bursting, seizure-like events, and depolarization block which are closely tied to the cubic-like nullcline geometry arising in Hodgkin–Huxley-type models, especially in the presence of slow ion dynamics.

Our work seeks to bridge biophysical realism with analytical tractability. The step-wise quadratic approximation we employ is specifically designed to mimic the cubic membrane potential profile that emerges from the full ion-exchange dynamics. While the Lorentzian Ansatz is not strictly justified in this case from first principles, we show that it yields a workable and biologically interpretable mean-field description, which aligns with single-neuron dynamics, population simulations, and even in vitro observations. To our knowledge, this is a novel contribution that extends mean-field modeling beyond currently available approaches, which are often restricted to simplified or phenomenological neuron models.

In this context, using a quadratic approximation is not merely a mathematical convenience — it is a means to retain key dynamical features of more realistic (non-Type I) neurons within a tractable framework, enabling insights into complex behaviors like multistability and pathological bursting.

(5) Sec. 1.2 As shown in Figure 3, the mean-field equations do not show a one-to-one correspondence with the neural network simulations, except in strongly synchronous regimes. This represents a strong limitation in the model, especially because exact neural mass models (as shown in Reference [23]) perfectly fit the dynamics of the underlying network model both in the asynchronous and in the synchronized regime.

We appreciate the reviewer’s observation and acknowledge that our original description may have caused confusion. The model's validity is not strictly limited to strongly synchronous regimes, but rather to regimes where the distribution of membrane potentials across the neuronal population remains unimodal and can be reasonably approximated by a Lorentzian. This includes but is not restricted to—highly synchronized states.

We agree that this distinction is important and have clarified it in the revised manuscript (e.g., “in strongly synchronous regimes” —> “in regimes where the membrane potentials' distribution is unimodal and can be reasonably approximated by a Lorentzian”).

In contrast to exact mean-field reductions based on quadratic integrate-and-fire neurons (e.g., [23]), our model originates from a biophysically grounded HH-type neuron with ion exchange dynamics, and necessarily involves heuristic approximations to achieve a closed-form mean-field description. While this results in a less exact correspondence with network simulations in more heterogeneous or bimodal states, our goal was to retain biological interpretability and account for phenomena such as ion-driven bursting and seizure-like transitions, which are not captured by standard QIF-based neural masses.

We see our contribution as complementary to existing exact reductions — offering a biophysically grounded alternative that remains tractable and informative in a relevant class of unimodal, mesoscopic dynamical regimes.

(6) Sec. 1.3 In this section the authors show the comparison between in vitro experiments and simulations with both the network model and the neural mass model (Figure 4, panels a,b,c). The qualitative agreement that is supposed to be shown is hardly evident. The shape of the signals is different as is the type of bursting. The only agreement results in the fact that there are repeated spiking events at successive times in a periodic manner. However, the time scale of the simulations is different for neural network simulation and mean-field experiment, making it difficult to compare them. While the period of the bursting event is around 2 min for mean field simulation (in according with experiments), the time scale of the network simulation is 60 times smaller, thus meaning that we are considering completely different mechanisms and phenomena. The justification given by the authors, that "the parameters were modified to simulate shorter fluctuations (in the network of Hodgkin-Huxley neurons) for computational efficiency" is inappropriate.

The poor agreement turns out to be even worse in the comparison between experiments and mean-field simulations shown in panels d and e of Figure 4. While the mean field simulation is characterized by a periodic behaviour both in the mean membrane potential and in the external potassium concentration, the in-vitro traces are not periodic and show an increasing irregular activity of the extracellular LFP in correspondence with increasing external potassium concentration.

How it is possible to justify the implementation of this model if the working hypotheses are not supported by the results? The worst agreement of the network simulations with the experiments reinforces the doubt raised in the previous point: what is the reasoning underlying the choice of Hodgkin-Huxley as a single neuron model?

We thank the reviewer for this detailed critique. We acknowledge that the comparisons in Figure 4 involve limitations and we now provide a clearer rationale and context in the revised manuscript. First, we emphasize that our intention is not to claim a quantitative match between the experimental and simulated traces, but rather to demonstrate that our model grounded in biophysical mechanisms such as ion exchange is capable of qualitatively reproducing a key feature observed experimentally: the slow modulation of neuronal activity by extracellular potassium concentration. For example, both in vitro (Fig. 4a, 4d) and in our simulations (Fig. 4b, 4e), bursts of activity ride on slower oscillations of potassium, and the interplay of fast and slow dynamics is central to both.

Regarding the discrepancy in timescales between the neural network and mean-field simulations: the network simulations were intentionally run with accelerated dynamics by rescaling biophysical parameters (e.g., membrane capacitance and gating time constants) to keep the computational cost feasible. We now clarify in the manuscript that this choice is standard practice in computational modeling when the primary goal is to validate dynamical mechanisms rather than replicate absolute timescales.

On the shape of LFP signals: the experimental recordings were AC-coupled, and the DC components associated with slower shifts in membrane potential such as those modeled in the mean-field simulations are not captured in those recordings. This limits the visibility of key features like the underlying potential jumps. Additionally, no claim is made regarding a specific bursting classification in either data or simulation.

We agree that the experimental trace in Fig. 4d shows more complex, non-periodic dynamics (e.g., slowing burst frequency and irregularity), which are not captured by our current deterministic model. These differences could plausibly arise from additional physiological processes (e.g., stochastic transitions between metastable regimes or variability in ion regulation) that are not modeled here. In future work, such phenomena may be captured by introducing noise or parameter variability (see, e.g., Saggio et al., A taxonomy of seizure dynamotypes , elife 2020), or by allowing the parabola coefficients in the nullcline approximation to vary dynamically.

Finally, regarding the choice of a Hodgkin–Huxley-type neuron: this model allows us to incorporate a biophysical description of ion exchange, which is central to the phenomena we study. While modeling the spiking mechanisms explicitly precludes certain mathematical simplifications available to very simplified neuron models with reset, it enables direct links between mesoscopic dynamics and measurable quantities such as extracellular potassium an essential objective of our work. To summarize, we rearranged Fig4:

Potassium can have periodic behavior with V bursting riding on top (Fig.4 a). The model also shows this behavior at different timescales (Fig. b,c,e).

AC LFP recording is filtered so we might not see the V jump during the bursts (because we do not have DC recordings). No claim about bursting class here.

Potassium can also have more complex behavior (e.g., slowing down of burst frequency Fig.4.d), that the deterministic model do not show, but maybe exploring dynamical parameters (e.g., from parabolas or K_bath) or with added noise allowing to jump between regimes (reference Saggio et al. eLife 2020).

(7) Sec. 1.5 Here six neural masses are coupled via long-range structural connections with random weights. Simulations of the system are shown for two different values of the global coupling parameter (G = 0 and G = 100). How many realisations of the network have been considered?

We thank the reviewer for pointing this out. The presented simulation was intended as a proof-of-concept demonstration to illustrate the model’s capacity to support network-level propagation of pathological activity. For this purpose, we considered a single representative realization of the structural connectivity with random weights. Given the deterministic nature of the model and the qualitative focus of the demonstration, additional realizations do not qualitatively change the observed behavior — namely, the transition from localized to network-wide bursting as coupling strength increases. We have now clarified this in the revised manuscript.

“This simulation serves as a proof of concept to illustrate how local pathological activity can propagate through a network depending on the strength of coupling. We used a single representative realization of randomly weighted structural connectivity. While we did not perform a systematic exploration of different realizations or coupling strengths, we observed that the qualitative behavior namely, the emergence of network-wide bursting beyond a critical coupling threshold remains robust across similar setups. The model is compatible with empirical connectome data and can be readily extended to simulations using realistic brain network architectures.”

In future applications involving data-driven network architectures or variability analyses, we agree that exploring multiple realizations or empirical connectomes will be valuable.

How do the results depend on the different choices of the random weights? What is the dependence of the emergent dynamics on G? What kind of dynamics can be observed varying smoothly the parameter G (e.g. from 0 to 100)?

This section serves as a proof of concept to show that pathological activity in one node can propagate through the network when coupling is strong. We used a single random weight configuration and did not systematically explore variations in G or connectivity. While richer dynamics likely emerge across intermediate values of G, a full parameter sweep is beyond the scope of this study. We clarify this in the revised text (see answer above).

(8) Sec. 2.1 In the description of the experiment it is mentioned that only Mg^{2+} is varied. What is the role played by Mg^{2+} variation in influencing the external potassium concentration variation? How the experiment can be linked to the model? How the hypothesis of introducing an equation for the potassium concentration current in the microscopic model is supported by the experiment and vice-versa?

We thank the reviewer for this question. We have added a new subsection in the Methods explaining the.agnesium removal as a mean to influence the external potassium dynamics:

“The membrane of hippocampal neurons is equipped with N-methyl-D-aspartate type glutamate receptors (NMDARs). These receptors have a very high affinity for glutamate and can, in principle, be activated by ambient glutamate present at low concentrations in the brain extracellular fluid (ECF). Under normal physiological conditions, this activation does not occur because extracellular magnesium ions (Mg^2+^) block the NMDAR channel at membrane potentials more negative than about –50 mV; this voltage-dependent block prevents receptor activation at rest. When extracellular magnesium is removed, the block is relieved, allowing NMDARs to be activated, leading to neuronal depolarization toward the action potential threshold [117].”

“In addition, as a divalent cation, Mg^2+^ interacts with the negatively charged neuronal membrane, contributing to the stabilization of the resting membrane potential. Lowering extracellular magnesium concentration disrupts this effect, resulting in membrane depolarization [118].”

“Consequently, magnesium removal not only facilitates NMDAR-dependent depolarization, but also directly depolarizes neurons. This depolarization increases the driving force for outward potassium currents through K^+^ channels, meaning that variations in Mg^2+^ can indirectly influence external potassium dynamics during neuronal activity.”

(9) Sec. 2.6 The modified version of the continuity equation has been derived following Reference [95], where the authors consider a network of Izhikevich neurons, and each neuron is modelled by a two-dimensional system consisting of a quadratic integrate and fire equation plus an equation that implements spike frequency adaptation. In particular, in [95] the authors achieve a closed set of mean-field equations with the inclusion of the mean-field dynamics of the adaptation variable by using a Lorentzian ansatz combined with the moment closure approach. The moment closure condition is also assumed in the present manuscript (Eq. 19). Under which assumptions is the implementation of the moment closure condition justified?

We are thankful to the reviewer (and also to the R2) for pointing out to the validity of the justification of the assumptions that we have used in our formalism. We hence agree that the moment closure is not a sufficient justification for assuming that V depends on the mean n, which is neccessary for the derivation of Eq. 20, but in addition we need the assumption that n can be treated as a collective variable as it is done in the works mentioned by the reviewer 2. In addition we have performed numerical simulations of the full system to calculate the error term introduced by this approximation, and the results in the new Fig. S2 show that this is below 2% for each of the different dynamical regimes.

We have hence modified the justification for Eq. (19) reading:

“Next we assume a first-order moment closure condition for the variable *n* [59], justified by the numerical simulations of the full network (see Fig. S2) which show that for most of the neurons (close to 99 \% for the value of ∆ same as in the other simulations) the mean of the population is well capturing the behavior of the single neurons [122]. Finally, putting together these factors and assuming that *n* can be treated as a collective variable for each neuron (see Limitations of the model} section) we arrive to ” and also

“The validity of the first moment closure, Eqs. (19), as in [59], is supported by the numerical simulations, which show that, both, during the silent regime and when seizure-like events occur, *n_i_* for most neurons track the network averaged ⟨*n* | V, η⟩. In particular, it is less than 2% of the neurons that fire while the mean is low, and vice-versa, Fig. S2. In less synchronized scenarios (larger ∆ or smaller *J*), however, this value would increase, but the mean would always capture the qualitative behaviour of the population.”

This is also now explicitly mentioned in the following paragraph:

“Unlike the mean membrane potential ⟨V⟩ and the firing rate (r), which can be explicitly derived from the continuity equation under the Lorentzian assumption, the expression for ⟨n(t)⟩ in Eq. (26) is formal. In our mean-field model, the gating variable (n) is treated as a global population variable, evolving deterministically as a function of the average membrane potential. Therefore, ⟨n(t)⟩ corresponds to the collective gating variable assumed to be shared by all neurons, and is not computed by averaging distinct microscopic (n_i_) values.”

(10) Considering also the comments reported above, I think that it would make more sense to start from an Izhikevich neuron model as microscopic model and add the equations for the ionic currents as mesoscopic variables (i.e. written as population average variables), instead of starting from the Hodgkin-Huxley single neuron model and trying to make hardly justifiable approximations and simplifications.

We respectfully disagree. While the Izhikevich model is computationally efficient, it lacks the biophysical detail required to capture key ion-driven mechanisms such as depolarization block, slow ion accumulation, and specific burst-initiation dynamics all of which are central to our study. The Hodgkin–Huxley framework, despite requiring approximation, provides the necessary physiological grounding to link microscopic ion exchange with emergent population behavior.

(11) Sec. 2.7 What is the advantage of using six more parameters to fit, like R-,R+,c-,c+,I-,I+?

This is in contradiction with the spirit of deriving a mean-field model, where the number of parameters should be reduced. What is the advantage of this mean-field derivation with respect to other mean-field derivations of Hodgkin-Huxley neurons, like the one in Reference [9]?

The additional parameters (R±, c±, I±) are not arbitrary they compactly parametrize the cubic-like nonlinearity of the membrane potential dynamics in our stepwise-quadratic approximation. This trade-off allows us to preserve essential biophysical features of HH neurons (e.g., bursting regimes, depolarization block) within a tractable analytic framework. Compared to alternative approaches like in ref. [9], which focus on phenomenological reductions and do not yield an ODE system, our model offers more direct interpretability in terms of ion dynamics, providing a closer link between microscopic mechanisms and mesoscopic activity patterns.

(12) Sec. 2.11 The derivation of the mean-field dynamics for the gating variable is rather heavy and difficult to follow. This section could be simplified, whilst also better explaining the underlying approximations and the validity of these approximations, which is currently missing.

We agree that the derivation is technical, but we chose to retain it for transparency, as it follows the Chen and Campbell approach and makes key approximations such as moment closure explicit. We have now added a clarification that n is treated as a collective variable We hope that the current level of detail helps readers understand the assumptions underlying the gating variable dynamics.

(13) Sec. 2.12 The derivation of Eqs. (36) is quite confusing and needs to be re-written in a clearer form. Why are both the variables x and r present in these equations, since they are proportional according to Eq. (25)?

We thank the reviewer for pointing this out. We have adjusted the equations to improve clarity and now consistently express the firing rate in terms of a single variable. This removes the redundancy and simplifies the presentation.

(14) Sec. 2.13 The derivation of Eqs. (37) is quite confusing and needs to be rewritten in a clearer form.

Both the auxiliary variable x and the firing rate r are present in this equation, the same as in Eq. (36). Therefore it is presented as a set of equations for the auxiliary variable x and for the physical variable V. Moreover in the equation for dV/dt, the quadratic term in V has disappeared and it is not clear to me which are the variables corresponding to I- and I+. In particular, in Eqs. (36) there are two different current terms I-,I+ for the two equations related to dy/dt. In Eqs. (37) there is a single term $(I_{cl} +I_{Na}+I_K+I_{pump})/C_m$ which is identical for both equations related to dV/dt. I was expecting two different terms also in Eqs. (37).

We appreciate the reviewer’s close reading. To improve clarity, we now express the dynamics in terms of the firing rate r, replacing $\dot{x}$\ with $\dot{r}$\ in both Eq. (36) and Eq. (37) to avoid confusion.

As for the current terms: in Eq. (37), we reverse the stepwise quadratic approximation and reintroduce the original ionic currents from Eq. (16). This is why the expressions involving $I_{\mathrm{cl}}, I_{\mathrm{Na}}, I_K, {\rm and}\, I_{\mathrm{pump}}$ appear as a single summed term in $\dot{V}$, rather than the split $I_-,I_+$ terms used in the stepwise approximation. We now clarify this in the text.

We also write V as $\bar{V}$ to clarify that it refers to the average membrane potential for the neuronal population. Finally, we wrote the final equation in a more compact form to improve clarity (new Eq.38).

(15) Moreover, while the equation for the gating variable n can be considered as a differential equation for a mesoscopic variable since n depends on average values only, it is not clear to me if the remaining variables $\Delta [K^{+}]_{\mathrm{int}},\quad [K^{+}]_{g}$ can be considered mesoscopic or not. Since Eqs. (37) represent a mean-field model, I expect every variable to be a mean-field variable. This could be easily achievable for the extracellular potassium concentration, but I do not understand how a site-specific microscopic variable like the intracellular potassium concentration variation can be automatically inserted in a set of mean-field equations without any averaging or intermediate steps. This is a crucial point to be clarified for the validity of the neural mass equations.

We thank the reviewer for raising this important point. In our model, we assume spatial homogeneity at the mesoscopic scale, meaning that ion concentrations — both intra- and extracellular — are uniformly distributed across the population. As a result, variables such as $\Delta[K^+]_{\mathrm{int}}, \Delta[K^+]_{\rm int}\, {\rm and}\, [K^+]g$ are treated as population-level averages, consistent with the mean-field framework.

Moreover, the rate of change of intracellular potassium is tightly coupled to extracellular dynamics via ion exchange mechanisms, justifying its inclusion as a slow, mesoscopic variable. We now clarify this modeling assumption explicitly in the text.

“By locally homogeneous, we mean that all neurons in the population are assumed to share the same extracellular and intracellular ionic environment and are connected with identical coupling rules, allowing us to treat the population as uniform with respect to ion dynamics and connectivity.”

“These slow variables are in addition considered to be mesoscopic, meaning they are identical for every neuron in the population.”

Minor points:

(1) Figure 2, panel d. Please detail the variable on the y-axis, which is not reported in the figure.

Done

(2) Eq. (15) is cited in many parts of the manuscript, while it seems to me it would be more appropriate to reference Eq. (2). Is this a mistake or is there a reason to cite Eq. (15)?

The reviewer is correct, we have had a wrong equation label, which we have now corrected.

(3) Figure 4 Would it be possible to show enlargements of the mean membrane potential traces to directly compare the different bursting types shown by the simulation of the different models?

The panel d already contains enlarged part of the membrane potential traces. For the rest, going back to the Q6, we want to stress again that our intention is not to claim a quantitative match between the experimental and simulated traces.

(4) Figure 5 In the caption the author refers to "the generic model, single neuron model, and epileptor model". Could you please better explain the models referred to and why they are mentioned? Are the generic model and the single neuron model those that are presented in the Materials and Methods section? Or do you refer to completely different models, as for the epileptor?

We have removed the reference to the generic model (we had in mind the canonical model for seizures by Saggio et al. 2017), since it is not mentioned in the paper, and we have clarified that the single neuron model and epileptor model, which were used to simulate seizure like events.

(5) Sec 2.5 As already stated above, the authors need to reduce unnecessary formulations that confuse the reader. Here, for example, Eqs. (6) and (7) are unnecessary, in view of the fact that delta spikes are used (Eq. 8).

We thank the reviewer for the suggestion, but we disagree, and we think it is better to start the derivations from the more general case, as done with Eqs. 6-7.

(6) Sec. 2.6 Could you please better explain why in Eqs. (15) and (16), the variable V0 is introduced, while before and after this, the variable V is used?

We thank the reviewer for the comment. In Eqs. (15) and (16), $\dot{V}_0$ denotes the free term of the membrane potential equation, i.e., the component driven solely by the intrinsic ionic currents and excluding the synaptic input I_syn_. Only this $\dot{V}_0$ term (a function rather than an independent variable) is approximated by the piece-wise quadratic expression in Eq.(21). In contrast, the variable V represents the membrane–potential variable, which dynamics is obtained by combining $\dot{V}_0$ with the synaptic current contribution I_syn_. In summary, there is no independent variable V_0_; only the function $\dot{V}_0$ is introduced to represent the intrinsic (non-synaptic) component of the membrane–potential dynamics. We have now clarified this in the text.

(7) In the square brackets of the r.h.s. of Eq. (18), for all the intermediate steps, it appears $G^{n}(V,n)\,\varrho^{V}$, while there should be $G^{n}(V,n)\,\varrho^{V}$.

We thank the reviewer for catching this typo. We have corrected this in the revised manuscript.

(8) Sec. 2.8 Here the authors affirm that "a double-Lorentzian (or a piece-wise Lorentzian) could be a suitable form for $\rho^{V}(t,V\mid\eta)$. However, it is not clear under which conditions such an assumption would allow a solution to the continuity equation". What are the problems underlying the implementation of the double Lorentzian? It seems to be a more correct form than the single Lorentzian actually implemented.

We thank the reviewer for this thoughtful question. In principle, a double-Lorentzian ansatz for $\rho^V$ can indeed be implemented in several reasonable ways–for example, by enforcing that the combined area of the two Lorentzian components is normalized to one (to preserve the probabilistic interpretation) and by imposing smoothness constraints at their boundaries. However, despite exploring these implementations, we were unable to obtain non-trivial solutions of the continuity equation under this parametrization. The only solvable case we found is the degenerate one in which the two Lorentzians collapse onto each other (i.e., $(x_- = x_+)\, {\rm and} \,(y_- = y_+)$), which reduces the ansatz to the single-Lorentzian form used in the manuscript. For this reason, although the double-Lorentzian is conceptually appealing, it did not yield practically useful solutions within our framework.

(9) Eq. (28). The symbols used for the flux (especially those used in the second-to-last step once the inner integration is performed) are confusing and it is difficult to understand what they mean.

We thank the reviewer for noting this issue. The problem was due to a LaTeX typo that prevented the vertical lines—indicating that the flux is evaluated at specific points—from rendering correctly. We have now corrected this.

(10) Eq. (29) In the third step there are some misprints that impair comprehension.

We thank the reviewer for noting this. We have corrected these misprints in the revised version.

(11) Line 696. The reference is not displayed.

Fixed.

**Reviewer #2 (Recommendations for the authors):**

As a really general remark, this manuscript is written in a confusing manner, the authors present their model in a general formulation and their analysis in a complicated way that in the end is not needed, as I will explain in detail in the following.

Another general question is why the authors want to employ the neural mass reduction methodology developed in [23] to obtain exact mean-field evolution for quadratic neurons (like the quadratic integrate and fire (QIF)) for a model that reveals a cubic dependence on the membrane potential, as the FizhHugh-Nagumo neuron (that indeed is a 2d reduction of the Hodkgin-Huxley model), to obtain an approximate neural mass model that somehow works qualitatively only for synchronized dynamics? Why not use another approach more suited to derive the neural mass model for cubic nonlinearity, as the one suggested in [33] and [69] by Di Volo and co-authors? What is the rationale behind the choice of the authors?

We appreciate the reviewer’s critical feedback and the opportunity to clarify our methodological choices. Our decision to base the mean-field model on Hodgkin–Huxley-type neurons stems from the need to retain ion channel dynamics, which are essential to capture the coupling between membrane activity and extracellular ionic concentrations. This biophysical link is central to our study and cannot be achieved using more abstract neuron models such as QIF or FitzHugh-Nagumo alone.

Regarding the mean-field reduction method: while the Ott-Antonsen/Lorentzian framework is indeed exact for QIF neurons, we adopted a stepwise quadratic approximation to apply a similar formalism to the cubic-like dynamics of the HH model. This choice enables us to analytically capture a rich set of behaviors, including bursting, depolarization block, and seizure-like dynamics, in a tractable mean-field system.

We considered the approach of Di Volo and colleagues [33, 69], but their methodology is tailored to asynchronous irregular regimes, whereas our model is specifically designed to capture dynamics in quasi-synchronous or bursting regimes — including epileptiform activity — which are not covered by the assumptions of the Di Volo framework.

We now clarify these modeling choices more explicitly in the revised manuscript.

"Unlike phenomenological or reduced models, the Hodgkin–Huxley framework allows us to retain explicit ion exchange dynamics, which are essential for linking membrane behavior to extracellular potassium fluctuations. This level of biophysical detail is crucial for modeling pathological regimes such as seizure onset and propagation."

Furthermore, the derivation of the neural mass equations is unnecessarily complicated, as a matter of fact, they approximate all the variables (except the membrane potentials of the single neurons) as collective variables (i.e. the gating variable and the potassium concentration) common to all the neurons. The neural network model for which they derive the neural mass model presents microscopic evolutions of the membrane potential cubic-like plus other global variables equal for all neurons, that depend on collective variables such as the mean membrane potential or the mean firing rate. Once clarified, the derivation of the neural mass model is much simpler, and it is not necessary to follow the approach reported in Reference [95] [Chen, L. & Campbell, S. A. Exact mean-field models for spiking neural networks with adaptation. Journal of Computational Neuroscience 50 (4), 445-469 (2022)] which is unnecessarily complicated. The authors can follow a much simpler methodology as explained by Guerriero et al in Reference [R6] (cited below) where the authors consider the same model studied in [95]. Such a methodology has been applied in many cases already, to introduce realistic aspects in the neural mass model [23] (see References [R1-R7] below). I strongly encourage the authors to reformulate their approach in a simpler and clearer manner, by following the approach reported in [R1-R7]. The manuscript will become more readable and it will gain in comprehension.

We thank the reviewer for this helpful suggestion. We agree that, given the assumptions made in our derivation (i.e., shared gating and ion concentration variables across neurons), the mean-field equations could alternatively be obtained using the simpler methodology proposed by Guerriero et al. [R6] and related works [R1–R7]. However, we chose to follow the derivation presented by Chen and Campbell [95] because it makes the approximations (e.g., moment closure, flux boundary assumptions) explicit and generalizable to future extensions. However, we also acknowledge that the assumption of n to be treated as a collective variable is needed, and for clarity, we have now added a remark in the manuscript indicating that the same result could be recovered more directly using the approach of Guerriero et al.

“We note that, under the assumption of globally shared gating and ion concentration variables across the neuronal population, the resulting mean-field equations can also be derived using simpler methods as proposed by Guerriero et al [58]. In this work, we follow the more general formalism of Chen and Campbell [59], which makes the role of key approximations (e.g., moment closure, vanishing flux at boundaries) explicit. This also facilitates potential generalizations to settings with partial heterogeneity or dynamic gating distributions.”

“Finally, putting together these factors and assuming that *n* can be treated as a collective variable for each neuron”

“Unlike the mean membrane potential ⟨V⟩ and the firing rate (r), which can be explicitly derived from the continuity equation under the Lorentzian assumption, the expression for ⟨n(t)⟩ in Eq. (26) is formal. In our mean-field model, the gating variable (n) is treated as a global population variable, evolving deterministically as a function of the average membrane potential. Therefore, ⟨n(t)⟩ corresponds to the collective gating variable assumed to be shared by all neurons, and is not computed by averaging distinct microscopic (n_i_) values.”

Now I will examine in detail all the manuscript and report comments/remarks/suggestions numbered as (Q#) on how to improve the present manuscript to render it easier to read and more comprehensible, these are not minor remarks, just detailed ones.

Introduction

(Q1) The Introduction section needs a part devoted to the reduction methodology developed in [23] for QIF neurons and a presentation of previous works dealing with the introduction of biologically realistic aspects in the neural mass model derived in [23]. Here is a non exhaustive list of such papers concerning the introduction of the following realistic aspects in the neural mass developed in [23]:

(I) short-term synaptic plasticity :

[R1] Exact neural mass model for synaptic-based working memory H Taher, A Torcini, S Olmi, PLOS Computational Biology 16 (12), e1008533 (2020)

[R2] Bursting in a next generation neural mass model with synaptic dynamics: a slow-fast approach H Taher, D Avitabile, M Desroches, Nonlinear Dynamics 108 (4), 4261-4285 (2022)

[R3] Mean-field approximations of networks of spiking neurons with short-term synaptic plasticity R Gast, K Thomas R, H Schmidt, Physical Review E 104 (4), 044310 (2021)

(II) spike frequency adaptation:

[R4] Gast, Richard, Helmut Schmidt, Thomas R. Knösche. "A mean-field description of bursting dynamics in spiking neural networks with short-term adaptation." Neural computation 32.9 (2020): 1615-1634.

[R5] Population spiking and bursting in next-generation neural masses with spike-frequency adaptation, A Ferrara, D Angulo-Garcia, A Torcini, S Olmi, Physical Review E 107 (2), 024311 (2023).

(III) conductance-based neuron with a slow current (Izekievic model):

[R6] A new generation of reduction methods for networks of neurons with complex dynamic phenotypes,IC Guerreiro, M Di Volo, B Gutkin, preprint arxiv: 2206.10370 (2022)

(IV) spike timing-dependent plasticity:

[R7] Mean-field approximations with adaptive coupling for networks with spike-timing-dependent plasticity, B Duchet, C Bick, Á Byrne, Neural computation 35 (9), 1481-1528 (2023).

(V) random connectivity and noise:

[R8] Mean-field models of populations of quadratic integrate-and-fire neurons with noise on the basis of the circular cumulant approach

DS Goldobin Chaos: An Interdisciplinary Journal of Nonlinear Science 31 (8) (2021)

[R9] A reduction methodology for fluctuation-driven population dynamics DS Goldobin, M Di Volo, A Torcini, Phys. Rev. Lett. 127, 038301 (2021)

[R10] Shot noise in next-generation neural mass models for finite-size networks VV Klinshov, SY Kirillov Physical Review E 106 (6), L062302 (2022)

I think the authors should refer in the introduction to these previous papers, where realistic biological aspects have been already introduced in the neural mass model developed in [23].

We have added a whole pragaraph devoted to the next-generation neural mass models and in particular to the other works introducing biological realism in this class of models:

“Recently, a class of these models, called next-generation neural mass models [42], has been developed based on an analytical approach introduced by [25] that allowed for the exact derivation of mean field parameters for a population of quadratic integrate-and-fire (QIF) neurons. These can be linked to EEG/MEG oscillations [43], including epipeltic seizures [44], and have been used to study various aspects of the whole-brain dynamics such as the low-dimensional manifold of the resting state [45, 46], aging [47] and neural sig natures of consciousness [48]. Number of works dealt with the introduction of biologically realistic aspects in the mostly phenomenological neural mass model derived in [25]. These included short-term synaptic plasticity [49–51], spike frequency adaptation [52, 53], spike timing-dependent plasticity [54], synaptic delay [29], random connectivity and noise [55–57], as well as an extension of the conductance-based neurons with a recovery variable [58–60].”

(Q2) Line 117 - Please specify what you mean by locally homogeneous, here.

Thank you for allowing us the opportunity to clarify this. We now report:

"By locally homogeneous, we mean that all neurons in the population are assumed to share the same extracellular and intracellular ionic environment and are connected with identical coupling rules, allowing us to treat the population as uniform with respect to ion dynamics and connectivity."

(Q3) In this sub-section the authors should clarify all the hypotheses they employ to derive the neural mass models, not only the Lorentzian approximation they did for a cubic model, but also the fact that they assume that the gating variable n is a global variable as well as that the potassium concentration are assumed to be the same for all neurons, that they assume no heterogeneity at this level. This is a fundamental aspect that should be clarified at this stage already.

We thank the reviewer for this important observation. We agree and have revised the text in the derivation section to explicitly state all key assumptions. Specifically, we now clarify that:

(1) The gating variable n is treated as a population-average (global) variable;

(2) The potassium concentrations Δ[K+]int and [K+]g are assumed to be homogeneous across the neuronal population; and (3) No heterogeneity is assumed at the level of the ion dynamics.

This assumption is biophysically motivated: ion concentrations — particularly extracellular potassium — tend to redistribute rapidly due to diffusion and electrochemical forces, leading to an effectively well-mixed environment at the mesoscopic scale. As such, assigning separate compartments to individual neurons is not justified in this modeling context. We now explicitly note this in the manuscript to avoid ambiguity.

“(3) We assume that the potassium concentrations, both intracellular $\left(\Delta [K^{+}]_{\mathrm{int}}\right)$ and extracellular (through the buffering variable $[K^{+}]_{g}$), are homogeneous across the neuronal population. This is justified physiologically by the rapid redistribution of ions through diffusion and electrochemical gradients, which enforce near-instantaneous equilibration at the mesoscopic scale. As such, assigning separate compartments to each neuron is neither practical nor biologically meaningful in this context. We assume that the potassium concentrations, both intracellular $\left(\Delta [K^{+}]_{\mathrm{int}}\right)$ and extracellular (through the buffering variable $[K^{+}]_{g}$), are homogeneous across the neuronal population. This is justified physiologically by the rapid redistribution of ions through diffusion and electrochemical gradients, which enforce near-instantaneous equilibration at the mesoscopic scale. As such, assigning separate compartments to each neuron is neither practical nor biologically meaningful in this context; (4) We assume that the gating variable n, which governs potassium conductance, can be treated as a population-averaged variable. This allows us to describe the neuronal ensemble using a reduced set of collective (mean-field) variables.”

Comparison with neural network simulations

(Q4) The comparison the authors perform between the microscopic model and the neural mass is misleading, From what the authors wrote it seems that you are considering 4 variables for each neuron in the network model (this is unclear from how the model is written in Eq (9)), I guess one for the membrane potential, one for the gating variable and two for the potassium concentration. However, this is not the network model for which the neural mass has been developed, the neural mass has been obtained for a network made of N + 3 variables (N membrane potentials and 3 collective variables for gate, and potassium concentrations) this is a sort of mesoscopic network models, analogously to what done previously in references [R1,R3,R4] above and others. If the authors would compare their neural mass with this mesoscopic model the agreement among the two would be improved.

We agree with reviewer’s observation and we now acknowledge this issue in the Results and in the Limitations. We have already modified the text to explicitly state that for the mean filed derivations n is treated as a collective variable and we have added the following statements:

“Also note that the gating variable *n* is treated as microscopic in the neural network, while in the derivations for the mean-field it is considered as a mesoscopic and identical for the whole population. This is likely responsible for some of the discrepancies between the two modalities.”

“Moreover, the discrepancy between the two modalities would have likely been smaller if for the neural network we also adopted a gating variable that is mesoscopic and identical across the spiking neurons, as in similar works [49–51]. However, here we demonstrate the validity of the mean-field approximation even for the more natural, microscopic representation of the gating variable in the neural network.”

Comparison with in vitro experiments

(Q5) Experiment -- The experiment is performed in vitro on the intact Hippocampus of mice between postnatal days P5-P7. It is known [R1] that neuronal activity at an early developmental stage is provided in the Hippocampus by a network primarily driven by synchronized GABA_A that provides an excitatory action and generates giant depolarizing potentials (GDPs) [R11]. However, GDPs have frequencies in the range of 1 Hz - 0.1 Hz, not matching the oscillation frequencies reported by the authors. I have several questions here:

(E1) At this stage P5-P7 are the interactions among neurons essentially excitatory? Or not, please explain why, Are the oscillations reported by the authors somehow related to GDPs? The depolarizing action of GABAergic transmission and the presence of GDPs during early rodent brain development, as described by Ben-Ari and some others researchers, are characteristics commonly observed in ex vivo brain preparations, but are not evident under physiological in vivo conditions (see doi: 10.3389/fphar.2012.00065).

In our preparation—intact mouse hippocampus—GABAergic synaptic transmission is not depolarizing. This is evidenced by the fact that inhibition of ionotropic GABA_A receptors with bicuculline triggers interictal-like discharges, which are routinely used as a model of epileptiform activity (see doi: 10.1016/j.nbd.2014.12.013). Therefore, in our experiments at P5–P7, neuronal interactions are not purely excitatory, and the observed low Mg2+ induced oscillations are not related to GDP.

(E2) What is the nature of the oscillations reported by the authors in Figure 4 ? Which is their origin, please explain in the text of the paper clearly.

The model of epileptic discharges presented in our study was first introduced over 20 years ago and has since become a well-established paradigm for screening potential antiepileptic drugs and research on the mechanism of epileptic seizure. A detailed description of this model can be found in doi: 10.1046/j.1460-9568.2002.02143.x, and its pharmacological properties are reviewed in doi: 10.1046/j.1528-1157.2003.19503.x. These references have now been added to the manuscript for clarity.

We have added the following:

“The model of epileptic discharges presented in our study was first introduced over 20 years ago [115] and has since become a well-established paradigm for screening potential antiepileptic drugs and research on the mechanism of epileptic seizure [116].”

(E3) How exactly does the concentration of extracellular potassium ions change, this is not clear even in Methods, please clarify.

[R11] Excitatory actions of GABA during development: the nature of the nurture Y Ben-Ari, Nature Reviews Neuroscience 3 (9), 728-739 (2002).

We have now added a new Subsection in the methods explaining how we use Mg2+ variation to influence the external potasium variation.

“The membrane of hippocampal neurons is equipped with N-methyl-D aspartate type glutamate receptors (NMDARs). These receptors have a very high affinity for glutamate and can, in principle, be activated by ambient glutamate present at low concentrations in the brain extracellular fluid (ECF).Under normal physiological conditions, this activation does not occur because extracellular magnesium ions (Mg^2+^) block the NMDAR channel at membrane potentials more negative than about –50 mV; this voltage-dependent block prevents receptor activation at rest. When extracellular magnesium is removed, the block is relieved, allowing NMDARs to be activated, leading to neuronal depolarization toward the action potential threshold [117]. In addition, as a divalent cation, Mg^2+^ interacts with the negatively charged neuronal membrane, contributing to the stabilization of the resting membrane potential. Lowering extracellular magnesium concentration disrupts this effect, resulting in membrane depolarization [118]”

“Consequently, magnesium removal not only facilitates NMDAR-dependent depolarization, but also directly depolarizes neurons. This depolarization increases the driving force for outward potassium currents through K^+^ channels, meaning that variations in Mg^2+^ can indirectly influence external potassium dynamics during neuronal activity.”

(Q6) Lines 187-191 and Figure 4 -- The authors wrote : "In Figure 4.c we show the membrane potential and external potassium for a simulation of N = 3000 coupled HH-like neurons showing a similar behavior, although the parameters were modified to simulate shorter fluctuations for computational efficiency." This sentence is unclear. What is clear from Figure 4 is that the network simulations gave rise to collective oscillations on a completely different scale seconds with respect to minutes and also the profile of the potassium concentration has a clearly different evolution. From Figure 4 one can conclude that network simulations have nothing to do with the neural mass evolution and the experiment. I think the authors should better clarify and describe the results reported in Figure 4.

We thank the reviewer for the observation. We have revised the relevant section of the manuscript to clarify the interpretation of Figure 4 and avoid any implication of quantitative matching. As stated in our response to Reviewer 1 (comment 6), the comparison is intended to highlight the shared qualitative structure across experimental data, the neural mass model, and the network simulation — specifically, the modulation of fast bursting by slow extracellular potassium fluctuations. The difference in timescale in the network simulation arises from rescaled parameters used for computational efficiency. We now explicitly state this and have updated the figure caption and accompanying text accordingly to reflect these points.

(Q7) Why do the authors consider a purely excitatory network to describe the experimental results? What is the reason for this choice? Why they do not consider as usual balanced excitatory- inhibitory networks? Please clarify this point.

We thank the reviewer for raising this point. We chose to model a purely excitatory network as a first step in isolating the role of extracellular potassium dynamics in generating population-level bursting. This allows us to focus on the ion-driven modulation mechanisms without introducing additional complexity from inhibitory feedback. Similar modeling choices have been made in previous studies of bursting and seizure-like dynamics (e.g., Gutkin et al.,), where inhibition is omitted to emphasize intrinsic or modulatory mechanisms. We acknowledge that incorporating inhibitory populations is an important next step for capturing a broader range of dynamics, but for the current study, the excitatory-only network provides a minimal and interpretable framework aligned with our focus.

(Q8) By comparing Figures 4 (a) and (b) it seems that the bursting activity observed in the experiment and in the mean-field simulations seem quite different, originating from different mechanisms and bifurcations, Can the authors comment on this?

We thank the reviewer for this important observation. We have reorganized the presentation of Figure 4 and revised the accompanying text to better clarify the nature of the comparison (see also our response to Reviewer 1, point 6). Our aim is not to claim that the experimental and simulated bursts arise from identical bifurcation mechanisms, but rather to highlight shared qualitative features — in particular, slow modulation of population activity by extracellular potassium. We now also comment on the potential role of more complex or noise-driven bifurcations (see Saggio et al. 2020) in shaping experimental bursting dynamics, which are not fully captured by the current deterministic model.

Bifurcation analysis: emergent network states and multistability

(Q9) This sub-section will gain interest by reporting simulations of the network and of the neural mass model presenting bistable dynamics.

We agree with the reviewer that this would be an important addition, but we believe that it goes beyond the scope of this work (for the computational reasons among others) and it remains for future work. We have however updated the bifurcation analysis section.

Limitations of the model

(Q10) Lines 276- 280 -- I think that the parameters c+,c_,R+,R_ depend not only on the slow variables, potassium concentrations but also on the actual value of the gate variable n. This should be stressed.

We thank the reviewer for this helpful observation. We agree and have clarified in the revised manuscript. This reflects the mean-field assumption that n is treated as a collective variable, and we now make this dependency explicit in the text.

“Furthermore, the parabola coefficients *c_-,c_+, R_-, R_+* were fixed as constants, however, these coefficients could be made functions of the slow variables and the gating variable, which might unveil new dynamical regimes and extend the validity of the thermodynamic limit beyond the regimes described in this work. Also, in the case of constant values, an in-depth exploration of the parameter space is required to fully characterize the model and its bifurcation structure.”

(Q11) The authors wrote: " Other limiting assumptions are the moment closure condition (19) and the assumptions that the functions (3) averaged across the neuronal population can be expressed as functions of the average membrane potential V and gating variable n which is only true in the cases where the functions (3) can be reasonably approximated as linear functions in a range of V and n." Apart from that a parenthesis is lacking, I think that this last aspect has been already taken into account when performing the fit with 2 parabolas to the sum of the currents, or not? In case, please specify.

We thank the reviewer for catching the missing parenthesis — this has been corrected in the revised manuscript. Regarding the modeling point: the two-parabola fit applies specifically to the membrane potential dynamics and captures the nonlinear dependence of the total current on V (eq.16). In contrast, the moment closure assumption involves approximating averages of nonlinear functions of both V and n, such as those appearing in the gating dynamics (e.g., n∞(V)). This is not directly accounted for by the parabola approximation, but is handled separately via the mean-field approximation of G^n as a function of the average variables (eq.15).

(Q12) A limitation that should be stressed is that the authors in the neural mass model consider the gate variable and the potassium concentrations, as global variable equal for all neurons, and where n depends on the mena membrane potential, to write that the moment closure (19) is a limiting assumption is honestly too clear, please be explicit here.

We have now the following two statements:

“These slow variables are in addition considered to be mesoscopic, meaning they are identical for every neuron in the population.”

“In our mean-field model, the gating variable (n) is treated as a global population variable, evolving deterministically as a function of the average membrane potential. Therefore, ⟨n(t)⟩ corresponds to the collective gating variable assumed to be shared by all neurons, and is not computed by averaging distinct microscopic (n_i_) values.”

Discussion

(Q13) The authors could discuss in this section the further biological ingredients they can introduce in their neural mass based on the previous works [R1-R9] that have already shown how to include plastic synapses, random connectivity, noise, adaptation, spike-timing-dependent plasticity, etc and which of these ingredients they consider more relevant for the whole brain dynamics.

In order not to repeat the same statements from the Introduction, we have now addded the following sentence:

“This approach, taking into account key biophysical details, offers a first step in considering the role of the glia in neural tissue excitability. Following this direction, other ions, such as calcium should be taken into consideration, as well as other effects such as plastic synapses, random connectivity, noise, adaptation, spike-timing-dependent plasticity, as already discussed in the Introduction.”

(Q14) The authors should also discuss why they limited their analysis to purely excitatory networks, and what would change by including excitatory-inhibitory interactions in each single mass and across neural masses, if this makes sense or not.

As stated in our response to Q7, we chose to focus on purely excitatory networks as a first step to isolate and study the core role of extracellular potassium dynamics in driving bursting behavior. This modeling choice allows for a minimal system where the interaction between intrinsic ionic mechanisms and network coupling is most transparent.

We also note that excitatory and inhibitory effects can be modeled within the same formalism by adjusting the synaptic reversal potential — for example, $E_{syn}=0$mV for excitatory, and $E_{syn}=-80$mV for inhibitory interactions. Including inhibitory populations would introduce additional complexity and richer dynamical regimes (e.g., oscillatory instabilities, balance states), which are certainly of interest but beyond the scope of this study.

Materials and Methods

(Q15) Fig.2 - I think a plus is lost in panel (c) where it should be [K+bath];

Thank you. We corrected the figure.

(Q16) Caption of Figure 2- the authors wrote: "In the case where the derivative of the membrane potential is zero for V > V ⋆ (e.g., if the cubic function is shifted up by adding a constant current to the membrane potential derivative), the population is described by the red distribution in the steady state, and the continuity equation is governed by the negative parabola equation." This sentence is unclear, the authors mean in the case where the derivative of the membrane potential crosses zero at V > V*? Please clarify.

We thank the reviewer for pointing this out. Yes, we refer to the case where the membrane potential derivative crosses zero at a point V>V∗. We have clarified this in the revised figure caption.

(Q17) Lines 558-562 -- Eqs (6) and (7) are examples of unnecessary complications of which this manuscript is full of. Since the authors do not consider any synaptic dynamics and homogenous (equal) couplings, these equations are not needed, I strongly recommend removing Eqs (6) and (7) and limiting to the expression reported in Eq (8), which indeed should also be corrected see next remark.

We appreciate the reviewer’s concern regarding clarity. As mentioned in our response to Reviewer 1, the inclusion of Eqs. (6) and (7) was intentional and serves a pedagogical purpose — to present the general structure of the network interactions before introducing simplifying assumptions. While we agree that Eq. (8) suffices for the simulations considered in this manuscript, we believe that showing the more general form helps clarify the model’s extensibility, for instance to cases with heterogeneous coupling or synaptic dynamics.

(Q18) Eq (8) - line 562 - Since the authors assume no synaptic evolution, i.e. instantaneous post-synaptic potentials, they can clarify that Eq (8) represents the population firing rate that later will be one of the fundamental variables of the neural mass model and call it r, as in the following. Furthermore, $s_i$ does not depend on the neuron index $i$ in a fully coupled network with homogenous coupling, as in the present case, this quantity is the same for all neurons. Please drop the index and call it r since it is the population firing rate.

We thank the reviewer for this useful suggestion. We now clarify in the text that under the assumptions of all-to-all homogeneous coupling and no synaptic dynamics, s_i is identical for all neurons and can be interpreted as the population firing rate r. This connection is made explicit in the revised manuscript.

“Under the assumption of instantaneous synaptic transmission and homogeneous all-to-all coupling, the synaptic activation variable (s_i_) is the same for all neurons and corresponds to the population firing rate, which we denote by (r)”

(Q19) Line 564-567 - Here the network model is incomplete, it is not sufficient that the authors report the evolution equation for the membrane potential Eq (9). They should report the evolution equation for the gate variable n and for the potassium concentration as done in Eq (1). This request is fundamental because it is unclear from the present formulation which are the variables that are microscopic (associated with the single neuron evolution) and which are global (common to all the neurons). This is a fundamental aspect and it should be clarified. I guess that n will depend on the neuron index $i$, while the potassium concentration it is unclear how the authors will consider them, global or local. I guess that the internal density should depend on the neuron index $i$ or not ? Anyway, I would like to know exactly which network model has been simulated e.g. to obtain the results reported in Figure 3.

We thank the reviewer for this essential clarification request. In the revised manuscript, we now explicitly state the full network model, including the evolution equations for the gating variable n_i and potassium variables. While in some simulations we consider the full microscopic model involving 4N variables (where each neuron has its own V_i ,n_i ,Δ[K+]int_i ,[K+]g_i), for the mean-field reduction and mesoscopic comparisons we assume that the gating and potassium variables are shared across neurons. This assumption is consistent with prior work (e.g., Chen & Campbell) and is biophysically justified in the case of potassium due to its fast spatial equilibration in extracellular space. We also now mention this explicitly in the Limitations.

(Q20) Continuity equation - Lines 568 - 597 - This part can be largely simplified and rewritten, as a matter of fact, the authors consider the gate variable n, the potassium concentrations as global (collective variables) depending on mean field values of they can directly start from eq 20, by stating that they assume that the other variables (n, $\Delta[K^+]_{int}$, $[K^+]_g$) are collective variables, common to all the neurons, and that depends only on mean field variables as or r. This has been done in many previous cases since the Ott-Antonsen Ansatz can be applied whenever the potential evolution is driven by quadratic terms and in the presence of mean field variables, the first indication of this was reported in 1993 by Watanabe and Strogatz for phase oscillators :

[R12] Watanabe, Shinya, and Steven H. Strogatz. "Integrability of a globally coupled oscillator array." Physical review letters 70.16 (1993): 2391.

Anyway, this approach has been previously employed to derive a neural mass model for networks of QIF neurons in the presence of various further neuronal variables (ranging from slow currents to plastic evolution of the couplings) describing more biologically realistic situations, see references [R1-R7] above. I strongly encourage the authors to reformulate their approach in a simpler and clearer manner, particularly interesting is for them the article [R6] by Guerriero et al, the authors examine exactly the same model as in Ref [95] [Chen, L. & Campbell, S. A. Exact mean-field models for spiking neural networks with adaptation. Journal of Computational Neuroscience 50 (4), 445-469 (2022)]. However, they solve the problem in a much more simple way, I encourage the authors to follow this approach.

We thank the reviewer for the constructive suggestion. We acknowledge that, under the assumption that n, Δ[K+]int , and [K+]g are collective variables shared across the neuronal population, one could directly begin from Eq. (20) and proceed using the simpler approaches found in Guerriero et al. [R6] or related works [R1–R7]. However, we chose to retain the Chen & Campbell formalism, with additional clarification regarding the mesoscopic nature of the gatin variable, as it explicitly highlights the key approximations used in the derivation, which may be beneficial for readers seeking to extend the method. See also general response to reviewer 2 at the beginning.

(Q21) Eq (26) -- I do not think the authors can estimate explicitly from the equation (26), as they do for the mean membrane potential and the firing rate. This is just a formal expression representing a collective variable, I do not think that will coincide with the average of the values of n_i for each neuron. Please discuss this point, and in this case show that indeed coincides with the average of all of the values of the single neuron gate variable n_i.

We thank the reviewer for raising this important point. We agree that Eq. (26) is more formal than operational, as ⟨n(t)⟩ is not directly derived from the continuity equation in the same way as ⟨V⟩ or the firing rate r. Rather, it reflects our mean-field assumption that the gating variable evolves as a collective population-averaged quantity, governed by the dynamics of the average membrane potential. In our formulation, n is treated as a global variable shared across neurons, and thus ⟨n(t)⟩ effectively is the gating variable in the neural mass model — rather than the result of averaging heterogeneous n_i. We have clarified this distinction in the text to avoid suggesting that Eq. (26) provides an explicit estimate of microscopic gating dynamics.

“Unlike the mean membrane potential ⟨V⟩ and the firing rate (r)>, which can be explicitly derived from the continuity equation under the Lorentzian assumption, the expression for ⟨n(t)⟩ in Eq. (26) is formal. In our mean-field model, the gating variable (n) is treated as a global population variable, evolving deterministically as a function of the average membrane potential. Therefore ⟨n(t)⟩ corresponds to the collective gating variable assumed to be shared by all neurons, and is not computed by averaging distinct microscopic (n_i_) values.”

(Q22) Mean-field dynamics for the gating variable - All this sub-section is in my opinion not useful, if the authors assume from the beginning that is a global variable. Indeed in the end they write for the evolution equation Eq (30) which is the same equation as for the single neuron gate variable (1) but for the mean values of n and . I suggest removing this sub-section.

We thank the reviewer for this suggestion. We agree that, under the assumption that n is a global collective variable, the resulting equation for ⟨n(t)⟩⟨n(t)⟩⟨n(t)⟩ is equivalent in form to the single-neuron gating equation, driven by the average membrane potential. However, we chose to retain this subsection to explicitly demonstrate how the gating dynamics enter into the mean-field formulation, especially for readers less familiar with this type of reduction. This step also mirrors the structure of the derivation used for other state variables in the model and maintains clarity for potential extensions where n may not be strictly global.

(Q23) Line 696 - here an equation reference is lost.

Thank you for pointing this out. We have corrected the text and restored the missing equation reference in the revised manuscript.

(Q24) Eqs (36) -(37) -- Since the variables r and x entered in Eq (36) are essentially the same as Eq (25), apart from a constant R/pi, the use of two different names complicated in a useless manner an already complicated expression, Please decide to use everywhere r or x and then proceed consequently this applies also to Eq (37). This will also allow us to rewrite the equation in x or r in a more compact form.

As noted in our response to Reviewer 1, point 14, we have revised Eq. (37) to ensure consistency in notation by replacing x with r throughout.

(Q25) Eq (37) - This equation is written in a manner that is not careful enough, apart from that the authors are passed now from (x,y) to (pi*r/R,V) , therefore they should substitute everywhere x with r. Furthermore, the equation for the derivative of V is confusing, the authors should use the same approximate expression employed in eq (36) that makes explicit the quadratic dependence on V itself, otherwise, I believe that the equation is incorrect.

In the same response to Reviewer 1, point 14, we also clarified the expression for $\dot{V}$ in Eq. (37), we reintroduced the full current-based formulation (as in Eq. 16), reversing the quadratic approximation used earlier. This is now explicitly stated in the text, and we have improved the equation presentation to avoid confusion.

(Q26) Eq (37) below line 708 - From this expression, it is clear that the gate variable n and the potassium variables are ruled exactly by the same equations as for the single neuron Eq (1) and that the Lorentzian Ansatz enter only in the rewriting of the evolution of the membrane potentials of the neurons in the network. In the end, the authors are doing exactly the same approximation made by many other authors [R1-R7], that these variables are collective, i.e. they are the same for all neurons, and in particular n=n(V) is a function of the mean membrane potential V. The mean field model that the authors derive corresponds to a microscopic model where the single neurons are heterogenous only in the intrinsic currents $\eta_i$, but they are all driven by collective variables, like n(V) and the potassium variables that are identical for all neurons. This should be clarified.

We agree with the conclusion by the reviewer, and as seen through the previous responses, we now explicitly acknowledge the fact that n and the two slow variables are considered as a mesoscopic variables for the mean-field derivation, while for the spiking network, n remains microscopic.
